# Response to letter regarding: Incidence, mortality, and DALYs of global pharyngeal cancer: systematic analysis and projections based on global burden of disease study 2021

**DOI:** 10.1080/07853890.2025.2564294

**Published:** 2025-09-22

**Authors:** Chen Xu, Tianjiao Zhou, Weijun Huang

**Affiliations:** ^a^ Department of Otorhinolaryngology Head and Neck Surgery, Shanghai Sixth People’s Hospital Affiliated to Shanghai Jiao Tong University School of Medicine, Shanghai, China; ^b^ Shanghai Key Laboratory of Sleep Disordered Breathing, Shanghai, China; ^c^ Otolaryngology Institute of Shanghai Jiao Tong University, Shanghai, China; ^d^ Department of Nursing, Shanghai Sixth People’s Hospital Affiliated to Shanghai Jiao Tong University School of Medicine, Shanghai, China

**Keywords:** Pharyngeal cancer, global burden of disease (GBD), estimated annual percentage change (EAPC), average annual percent change (AAPC), trend analysis

Dear Editor

We sincerely thank Dr. Wang and colleagues for their careful reading of our article and for their thoughtful and constructive comments regarding the trend analysis methodology. We greatly appreciate their attention to the distinction between estimated annual percentage change (EAPC) and average annual percent change (AAPC), which are both important indicators for epidemiological trend assessment.

As they rightly pointed out, EAPC offers a simple and intuitive approach by fitting a single log-linear regression to age-standardized rates over the entire observation period [1]. This makes it particularly suitable for summarizing long-term, overall patterns of disease burden change. Nevertheless, EAPC assumes a constant linear trend, which may obscure potential turning points or phase-specific changes and may be influenced by extreme values at the beginning or end of the time series.

In contrast, AAPC is calculated using Joinpoint segmented regression and provides a time-weighted average of annual percentage changes across statistically defined segments [2]. This method has the advantage of capturing nonlinear patterns, identifying inflection points, and reflecting recent trends more accurately, thus enhancing policy relevance. However, AAPC requires additional modeling steps, relies on specialized software, and its interpretation may be less straightforward for a general readership.

Our primary objective in this study was to present macro-level global and regional trends in pharyngeal cancer burden over a 30-year period (1990–2021) [3]. For this purpose, EAPC was selected because it offers a parsimonious, consistent, and comparable measure across 204 countries and territories, allowing clear visualization of long-term trends. Including segmented regression and AAPC calculations for every country and outcome would have significantly increased the computational burden and complexity of interpretation, potentially detracting from the clarity and accessibility of the results.

Nevertheless, we fully agree that AAPC can complement EAPC by providing additional insight into recent temporal changes. Future studies focusing on short-term dynamics, turning points, or the effects of specific public health interventions could greatly benefit from incorporating AAPC to provide a more nuanced understanding of epidemiological patterns.

Once again, we thank Dr. Wang and colleagues for their valuable suggestions, which will inspire methodological refinements in our future research.

## Data Availability

Data availability is not applicable to this article as no new data were created or analyzed in this study.
